# Inhibition of Hedgehog Signaling in Fibroblasts, Pancreatic, and Lung Tumor Cells by Oxy186, an Oxysterol Analogue with Drug-Like Properties

**DOI:** 10.3390/cells8050509

**Published:** 2019-05-27

**Authors:** Feng Wang, Frank Stappenbeck, Farhad Parhami

**Affiliations:** MAX BioPharma Inc., 2870 Colorado Avenue, Santa Monica, CA 90404, USA; fwang@maxbiopharma.com (F.W.); fstappenbeck@maxbiopharma.com (F.S.)

**Keywords:** cancer, hedgehog signaling, non-canonical activation of Gli, oxysterols

## Abstract

The widespread involvement of the Hedgehog (Hh) signaling pathway in human malignancies has motivated the clinical development of Smoothened (Smo) antagonists, such as vismodegib and sonidegib. However, Smo antagonists have failed to benefit patients suffering from Hh pathway-dependent solid tumors, such as pancreatic, colorectal, or ovarian cancer. Hh-dependent cancers are often driven by activating mutations that occur downstream of Smo and directly activate the transcription factors known as glioma-associated oncogenes (*Gli1-3*). Hence, the direct targeting of Gli could be a more effective strategy for achieving disease modification compared to Smo antagonism. In this study, we report on the biological and pharmacological evaluation of Oxy186, a semisynthetic oxysterol analogue, as a novel inhibitor of Hh signaling acting downstream of Smo, with encouraging drug-like properties. Oxy186 exhibits strong inhibition of ligand-induced Hh signaling in NIH3T3-E1 fibroblasts, as well as in constitutively activated Hh signaling in Suppressor of Fused (Sufu) null mouse embryonic fibroblast (MEF) cells. Oxy186 also inhibits Gli1 transcriptional activity in NIH3T3-E1 cells expressing exogenous Gli1 and Gli-dependent reporter constructs. Furthermore, Oxy186 suppresses Hh signaling in PANC-1 cells, a human pancreatic ductal adenocarcinoma (PDAC) tumor cell line, as well as PANC-1 cell proliferation in vitro, and in human lung cancer cell lines, A549 and H2039.

## 1. Introduction

**Figure 1:**
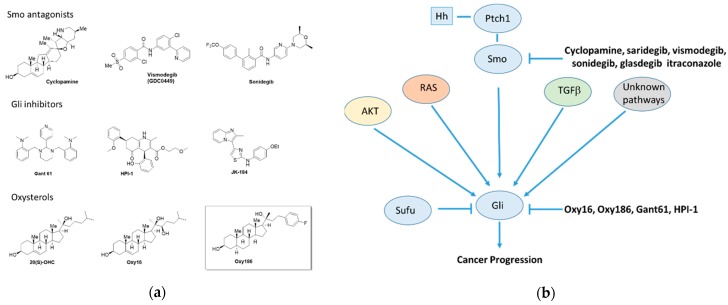
(**a**) Small molecule modulators of Hedgehog signaling and (**b**) canonical and non-canonical regulation of GLI and Hedgehog signaling.

Indispensable for cell fate decisions and required for proper tissue and organ development, the Hedgehog (Hh) signaling pathway commences activity during embryonic development and remains activated throughout the prenatal period [1]. In the adult organism, by contrast, Hh signaling lies mostly dormant, except for brief bursts of localized signaling during maintenance of stem cell populations, homeostasis, and regeneration of tissues [2,3,4]. Aberrant postnatal activation of Hh signaling is broadly linked to many forms of human cancer and often implicated in critical aspects of carcinogenesis, disease progression, and metastasis. Activation of Hh signaling in cancer can occur via several mechanisms, such as ectopic production of Hh ligands, somatic mutations in the Hh pathway components, *Ptch1*, *Smo*, *Sufu*, and/or amplifications of the Hh transcription factor Gli1 [5]. In addition, non-canonical Hh signaling, defined as Smo-independent Gli1 activation, can be activated by various branches of the TGFβ, EGFR, Ras-Erk, and PI3K-Akt-mTOR signaling pathways (Figure 1b) [5,6,7,8,9]. PDAC, a devastating disease with a particularly poor prognosis, and non-small cell lung cancer (NSCLC), the leading cause of cancer-related deaths worldwide, stand out among solid tumors often associated with inappropriate Hh pathway activation, which also include brain, breast, colorectal, gastric, liver, ovarian, and prostate cancers [10]. 

In the search for new drugs targeting this pathway, numerous small molecules have been characterized as modulators of Hh signaling both in vitro and in vivo, such as the plant-derived alkaloid cyclopamine, the related drug candidate saridegib, FDA approved cancer drugs vismodegib, sonidegib, and glasdegib, and various oxysterols and other molecules, such as itraconazole (Figure 1a,b). All clinically approved Hh pathway inhibitors to date (vismodegib, sonidegib, and glasdegib) are inhibitors of the G protein-like coupled receptor Smoothened (Smo), so called Smo antagonists [11]. These drugs are most effective in tumors driven by constitutively activated Hh signaling due to somatic mutations at the level of Patched (*Ptch1*), as frequently encountered in basal cell carcinoma (BCC) of the skin, which can be quite effectively treated with Smo antagonists [12]. Unfortunately, efforts to extend the clinical applications of Smo antagonists beyond BCC, such as in pancreatic, colorectal, or ovarian cancer, have been mostly unsuccessful to date [13,14,15,16]. However, glasdegib, a recently approved drug developed by Pfizer, has shown promise in clinical trials for acute myeloid leukemia [17]. 

**Figure 2:**
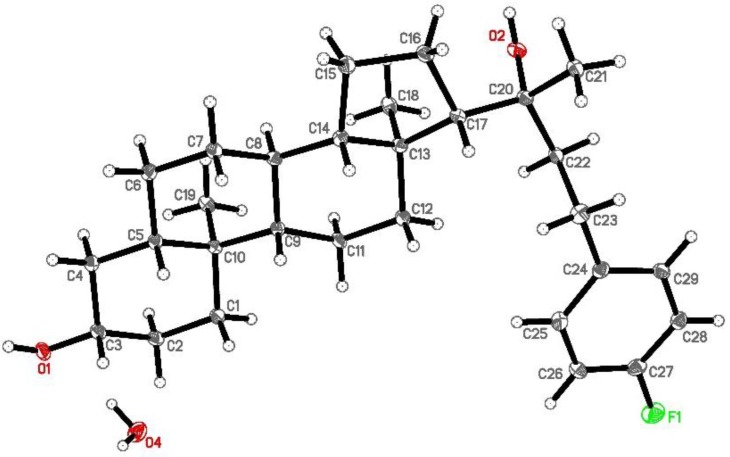
Ortep representation of Oxy186 in the solid state.

**Scheme 1:**
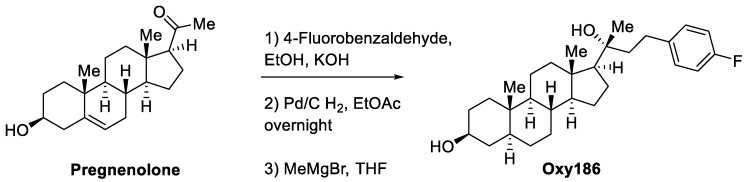
Synthesis of Oxy186.

Many Hh-dependent cancers are driven by activating mutations that occur downstream of Smo, affecting the transcription factors known as glioma-associated oncogenes (*Gli1–3*), which mediate the transcriptional effects of Hh pathway activation [18]. Numerous studies point to the significance of Gli activity in tumor cells, as well as the tumor microenvironment, particularly with stroma rich tumors, such as PDAC [19,20,21]. Inappropriate Hh signaling has been reported to occur at least in part via a paracrine signaling mechanism in which the expression and secretion of Hh ligand by tumor cells entails the subsequent activation of the canonical Hh signaling in adjacent stromal cells [22,23], although the role of paracrine Hh signaling in the pathogenesis of cancer has become a controversial topic [24,25]. Other studies have demonstrated that both mutant KRAS and TGFβ pathway signaling can also induce Gli activation in a non-canonical and Smo independent manner [26,27,28,29]. Therefore, new drugs acting at or near the level of the Gli transcription factors could significantly broaden the clinical utility of Hh pathway inhibition and such inhibitors would also be beneficial in settings with ligand-induced signaling. Pioneering studies in Gli inhibition have identified Gant61, HPI-1, and JK-184 (Figure 1a) as lead molecules capable of Hh pathway inhibition ‘South of Smo’; however, none of these leads has progressed into clinical testing [30,31,32,33]. In the current study, we report on the biological and pharmacological evaluation of oxysterol analogue Oxy186 (Figure 2, Scheme 1), a novel inhibitor of Hh signaling acting downstream of Smo with encouraging drug-like properties, including metabolic stability, oral bioavailability in mice, and chemical scalability through facile low-cost synthesis. 

## 2. Materials and Methods

### 2.1. Synthesis and Molecular Characterization of Oxy186

Materials were obtained from commercial suppliers and were used without further purification. Air- or moisture-sensitive reactions were conducted under an argon atmosphere using oven-dried glassware and standard syringe/septa techniques. The reactions were monitored on silica gel TLC plates under UV light (254 nm) followed by visualization with Hanessian’s staining solution. Chromatographic purifications were performed using a Teledyne ISCO CombiFlash Rf automated chromatography system. NMR spectra were measured in CDCl_3_. The data are reported as follows in ppm from an internal standard (TMS, 0.0 ppm): chemical shift (multiplicity, integration, coupling constant in Hz.).

*(3S,5S,8R,9S,10S,13S,14S,17S)-17-((R)-4-(4-fluorophenyl)-2-hydroxybutan-2-yl)-10,13-dimethylhexadecahydro-1H-cyclopenta[a]phenanthren-3-ol* (Oxy186): Oxy186 was prepared in three synthetic steps as depicted in Scheme 1. Briefly, pregnenolone was condensed with 4-fluorobenzaldehyde to the enone, which was reduced along with the C-5,6 double bond by hydrogenation using palladium on carbon (Pd/C) as a catalyst. The resulting fully saturated ketone was reacted with methylmagnesium bromide to afford the 20(*R*)-tertiary alcohol, Oxy186, as predicted by the Felkin-Ahn model [34] and verified by us via single crystal x-ray crystallography. The crude product was purified by chromatography on silica. ^1^H NMR (CDCl_3_, 400 MHZ) δ: 7.14–7.11 (2H, m), 6.97 (2H, dd, *J* = 8.8, 8.8 Hz), 3.54 (1H, dddd, *J* = 0.9,10.9, 5.5, 5.5 Hz), 2.73–2.64 (2H, m), 2.32–1.22 (15H, m), 1.21 (3H, s), 0.80 (3H, s), 0.76 (3H, s). ^13^C NMR (CDCl_3_, 100 MHZ) δ: 161.2 (d, *J* = 242 Hz), 138.2 (d, *J* = 3.1), 129.6 (d, *J* = 20 Hz), 115.1 (d, *J* = 20 Hz), 75.7, 71.3, 58.8, 56.7, 54.3, 44.9, 44.7, 43.3, 40.6, 38.15, 37.0, 35.5, 34.9, 32.0, 31.5, 29.6, 28.7, 23.8, 26.8, 23.7, 23.3, 21.1, 14.0, 12.3. MS (ESI-ve): [M – H] = 441.2 conforms to structural formula C29H43FO2, MW = 442.32. A 5 mg portion of Oxy186 was dissolved in EtOH (0.5 mL) and crystallization was induced by slow evaporation of the solvent. Single crystal X-ray diffraction data were collected at 100 K on a diffractometer with Bruker Apex-II CCD detector and a Cu-micro focus source. Crystal data: Orthorhombic, a = 7.26680(10) Å, b = 13.1667(2) Å, c =26.4392(5) Å, α= 90° β = 90°, γ = 90°, Vol. = 2529.70(7) Å3, Space group = P212121. The final anisotropic full matrix least-squares refinement on F2 converged at R1 = 0.0345, wR2 = 0.078, and GOF = 1.04.

### 2.2. Cell Culture and Reagents

NIH3T3-E1 fibroblasts were obtained from ATCC (Manassas, VA) and cultured, as previously described [35,36]. CAPAN-1 and PANC-1 human pancreatic cancer cells were obtained from ATCC and cultured in Dulbecco’s Modified Eagle Medium (DMEM) containing 10% heat-inactivated fetal bovine serum (FBS) (Hyclone Laboratories, Logan, UT, USA) and antibiotics, as previously described [37]. The human lung cancer cell lines A549 and H2030 were obtained from ATCC and cultured in RPMI-1640 containing 10% FBS. The human hepatoma cell line HepG2 was obtained from ATCC and cultured in DMEM containing 10% FBS. Mouse embryonic fibroblasts (MEFs) from suppressor of fused (*Sufu*) null mice (*Sufu*^−/−^) were provided by Dr. Philip Beachy of Stanford University and cultured in DMEM containing 10% FBS. Gant61, SB431542 and vismodegib (GDC0449) were obtained from Cayman Chemical, HPI-1 was obtained from Abcam. For experiments, cells were treated in a medium containing 5% FBS.

### 2.3. CAPAN-1 Conditioned Medium

Growth medium in confluent 10 cm^2^ tissue culture plates of CAPAN-1 cells were replaced with 10 ml of fresh growth medium and cells were incubated for a total of 7 days. Conditioned medium (CM), containing Shh and Ihh, was collected and spun down to remove any detached cells and debris, aliquoted, and stored frozen at −80 °C [37].

### 2.4. Quantitative RT-PCR

Total RNA was extracted with the RNeasy Plus Mini Kit from Qiagen, according to the manufacturer’s instructions. One microgram of RNA was reverse-transcribed using an iScript Reverse Transcription Supermix from Bio-Rad, to make single-stranded cDNA. The cDNAs were then mixed with Qi SYBR Green Supermix (Bio-Rad, Hercules, CA, USA) for quantitative RT-PCR assay using a Bio-Rad I-cycler IQ quantitative thermocycler. All PCR samples were prepared in triplicate wells in a 96-well plate. After 40 cycles of PCR, melt curves were examined in order to ensure primer specificity. Fold changes in gene expression were calculated using the ΔΔCt method. Primers used for mouse were as follows: *Oaz1* (5’-CCACTGCTTCGCCAGAGAG-3’) and (5’-CCCGGACCCAGGTTACTA-3’), *Gli1* (5’-GCTTGGATGAAGGACCTTGTG-3’ and 5’-GCTGATCCAGCCTAAGGTTCTC-3’), *Ptch1* (5’-CCATCGGCGACAAGAACC-3’ and 5’-CCAGCACAGCAAAGAAATACC-3’), *Hip* (5’-GGCTCTGTCGAAACGGCTACTAC-3’ and 5’-GCACGCTGGCTCACACTTGG-3’), and *Abca1* (5’-TGCCACTTTCCGAATAAAGC-3’ and 5’-GGAGTTGGATAACGGAAGCA-3’). Primers used for human were as follows: *SREBP1c* (5’-CGCTCCTCCATCAATGACA-3’ and 5’-TGCGCAAGACAGCAGATTTA-3’), *GAPDH* (5’-CCTCAAGATCATCAGCAATGCCTCCT-3’ and 5’-GGTCATGAGTCCTTCCACGATACCAA-3’), *SHH* (5’-CGGAGCGAGGAAGGGAAAG-3’) and (5’-TTGGGGATAAACTGCTTGTAGGC-3’), and *GLI1* (5’-GAAGCCGAGCCGAGTATC-3’ and 5’-GGTGAGTAGACAGAGGTTGG-3’).

### 2.5. Transient Transfection Assay

NIH3T3-E1 and Sufu^−/−^ cells cultured in 24-well plates at 90% confluence were transiently transfected with 0.1 µg of Gli response-element reporter (pGL3b-8xGli-Luciferase) plasmid, and 10 ng of pTK-Renilla-Luciferase plasmid with or without co-transfection with 10 ng of Gli1 overexpression vector, pSRα-Gli1, as previously described using Lipofectamine LTX Plus transfection reagent (Invitrogen) [38]. Twenty-four h after transfection, cells were treated with test agents for 72 h. Then the firefly and Renilla luciferase activities were measured using a dual luciferase kit (Promega, Madison, WI, USA) and a GloMax-96 Microplate Luminometer. The firefly luciferase activities were normalized to the Renilla luciferase activities. Data are reported as the mean of triplicate determinations ± SD.

### 2.6. Cell Counting Assay

A549 and H2030 cells cultured in 12-well plates at 20% confluence were treated with Oxy186, HPI-1, Gant61, or GDC0449 for 96 hours and then trypsinized and suspended in fresh medium. An aliquot of cell suspension was applied to a hemocytometer and counted under a microscope.

### 2.7. Statistical Analysis

Statistical analyses were performed using the StatView 5 program (SAS Institute, Cary, NC, USA). All *p* values were calculated using ANOVA and Fisher’s projected least significant difference (PLSD) significance test. A value of *p* < 0.05 was considered significant.

## 3. Results

In a previous study, we demonstrated that Hh signaling activated in fibroblastic cells by Hh proteins produced by CAPAN-1 pancreatic tumor cells can be suppressed in the presence of Oxy16 (20-α, 22(R)-dihydroxycholesterol), a naturally occurring oxysterol and metabolite of cholesterol [37]. This assay represents a simplified in vitro model of ligand-activated Hh signaling that may occur in PDAC stroma and molecules, such as Oxy16, that can inhibit the signaling and can be considered as possible starting points in the development of new drugs that target aberrant Hh signaling in PDAC-associated stroma and PDAC tumor cells. Using this assay, we screened about 70 structural analogues of Oxy16 synthesized in our laboratory and identified Oxy186 as a superior semisynthetic analogue, with improved physicochemical and absorption, distribution, metabolism, and excretion (ADME) properties. As Oxy16 is difficult to obtain from natural sources, it must be prepared according to a published synthesis protocol in six steps that include tedious chromatographic separations [39]. By contrast, Oxy186 can be readily prepared in three simple steps on a multi gram scale using inexpensive starting materials, such as pregnenolone, 4-fluorobenzaldehyde and methylmagnesium bromide (Scheme 1). 

### 3.1. Oxy186 Inhibits Hh Signaling in Mouse Fibroblasts and Human Cancer Cells

**Figure 3:**
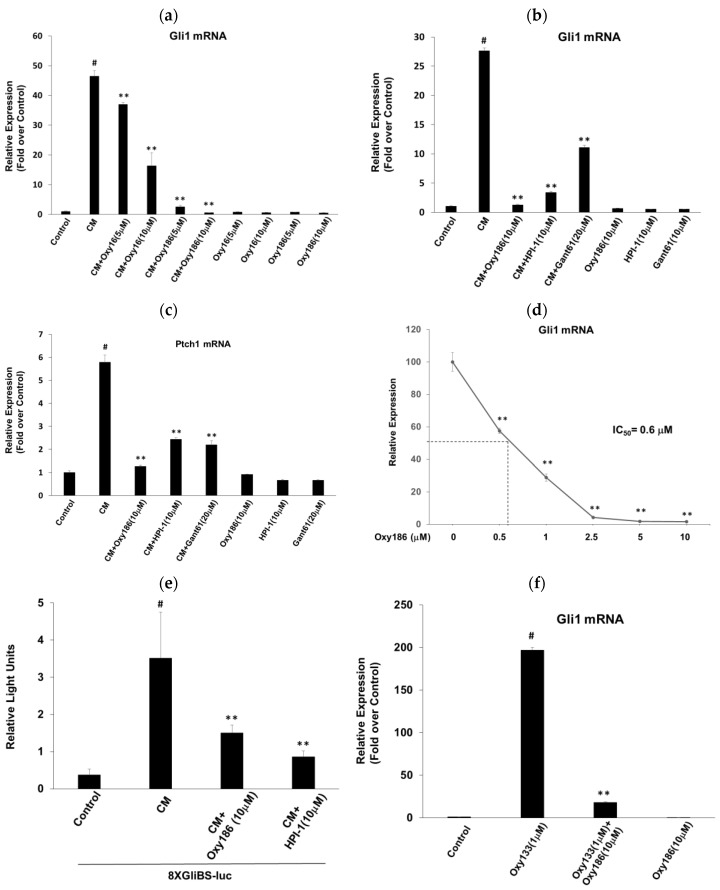
Inhibition of ligand-induced Hedgehog signaling. NIH3T3 cells were pretreated for 2 h with the oxysterols or various inhibitors, as indicated, in DMEM containing 5% FBS. Next, cells were treated with CAPAN-1 conditioned medium (CM) in the absence or presence of oxysterols or inhibitors. After 72 h, RNA was extracted and analyzed by Q-RT-PCR for the expression of Hh target genes *Gli1* (**a**,**b**,**d**) and *Ptch1* (**c**), and normalized to *Oaz1* expression. (**e**) NIH3T3 cells cultured in 24-well plates were transfected with a Gli response-element reporter (pGL3b-8xGliBS-Luciferase) plasmid and a pTK-Renilla-Luciferase plasmid. 24 h after transfection, cells were treated with the test agents, as indicated for 72 h. Luciferase activity was measured and normalized to the Renilla luciferase activity. (**f**) NIH3T3 cells were treated with the oxysterols, as indicated, in DMEM containing 5% FBS for 72 h. Q-RT-PCR was performed as described in (**a**). Data from a representative experiment are reported as the mean of triplicate determinations ± SD (** *p* < 0.01 vs. CM-treated cells (**a**–**e**) or Oxy133 (**f**); ^#^ *p* < 0.01 vs. control).

We examined the effects of Oxy186 on Hh signaling in NIH3T3-E1 mouse fibroblastic cells treated with CAPAN-1 conditioned medium (CM), which we previously reported to contain Hh ligands [37], and found that it robustly inhibited the mRNA expression of ligand-induced Hh target genes *Gli1* (Figure 3a,b) and *Ptch1* (Figure 3c). Under these experimental conditions, Oxy186 exhibited greater Hh signaling inhibitory efficacy than Oxy16, comparable with established Gli1 inhibitors, HPI-1 (10 µM) and Gant61 (20 µM) (Figure 3b,c), with Oxy186 displaying a submicromolar IC_50_ (0.6 µM) for inhibition of Gli1 expression (Figure 3d). To further validate the inhibitory effect of Oxy186 on Hh signaling, we performed a luciferase reporter assay and found that Oxy186 and HPI-1 significantly inhibited the CM-induced activity of a 8XGliBS-luciferase reporter that is responsive to Gli1 in NIH3T3-E1 cells (Figure 3e). In addition to ligand-induced Hh signaling, Oxy186 significantly suppressed Hh signaling that resulted from direct allosteric activation of Smo, as shown in Figure 3f, mediated by oxysterol Oxy133, a known allosteric modulator of Smo (38), confirming the inhibitory effects of Oxy186 on Smo mediated activation of Hh signaling.

**Figure 4:**
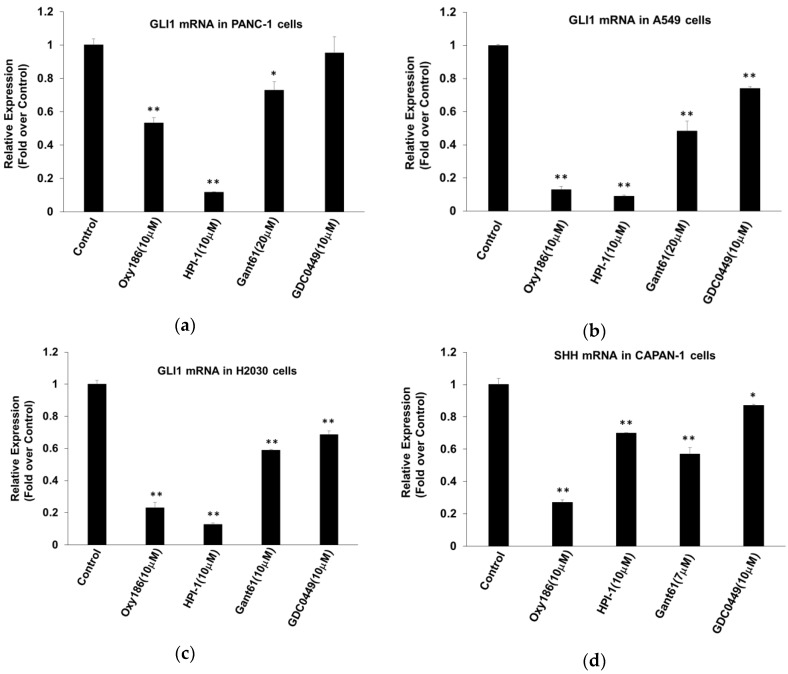
Inhibition of *Gli* and *Shh* expression in (**a**) PDAC (PANC-1) and (**b**,**c**) human lung cancer cells (A549 and H2030, respectively) by Oxy186. Cells were treated with the compounds as indicated in DMEM or RPMI 1640 containing 5% FBS for (**a**) 48 or (**b**,**c**) 72 hours, then RNA was extracted and analyzed by Q-RT-PCR for the expression of *Gli1* and normalized to *GAPDH* expression. (**d**) CAPAN-1 cells were treated with the compounds, as indicated, in DMEM containing 5% FBS for 72 h, then RNA was extracted and analyzed by Q-RT-PCR for the expression of *Shh* and normalized to *GAPDH* expression. Data from a representative experiment are reported as the mean of triplicate determinations ± SD (* *p* < 0.05 vs. control; ** *p* < 0.01 vs. control).

Gli1 transcription factors are the effectors of Hh signaling. It has been demonstrated that Gli1 activity in many human malignancies, including PDAC, is required for tumorigenesis and that both mutant KRAS and TGFβ signaling can induce Gli activity in a Smo-independent manner [27,28,29]. Accordingly, direct inhibition of Gli by siRNA or by the Gli inhibitor Gant61 can block growth and survival of pancreatic cancer cells [40]. Experimental evidence also shows Gli1 involvement in both small- and non–small-cell lung cancers (SCLC and NSCLC) [10,41]. Direct inhibition of Gli, perhaps in combination with other chemotherapeutic agents, therefore, could represent a promising strategy for the treatment of different malignancies. Treatment of PANC-1 cells with Oxy186 at 10 µM, or with Gli inhibitors HPI-1 (10 µM) or Gant61 (20 µM), significantly inhibited the high baseline expression of *Gli1* [27], whereas treatment with the Smo antagonist vismodegib (GDC0449) (10 µM) did not have a similar effect (Figure 4a). Treatment of A549 and H2030 cells with Oxy186 at 10 µM, or with Gli inhibitors HPI-1 (10 µM) and Gant61 (20 µM), or with the Smo antagonist GDC0449 (10 µM), significantly inhibited the expression of *Gli1* (Figure 4b,c). 

Tumor cells have been shown to secrete Hh ligands that may stimulate tumor stromal cells to produce various factors that facilitate growth and metastasis of the tumor cells [7,42]. This type of paracrine Hh signaling emanating from tumor cells is believed to play an important role in tumor growth and dissemination in several solid tumors, including PDAC [7,22]. We previously reported that CAPAN-1 pancreatic cancer cells produce Hh ligands [37], and, in the present study, we examined the ability of Oxy186 to inhibit the expression of *Shh* in these cells. Our results showed that Oxy186 robustly inhibited *Shh* expression in CAPAN-1 cells (Figure 4d). HPI-1, Gant61, and GDC0449 also had inhibitory effects on Shh expression in CAPAN-1 cells, but to a lesser extent (Figure 4d). 

### 3.2. Oxy186 Inhibits Hh Signaling Epistatic to Sufu^−^

**Figure 5:**
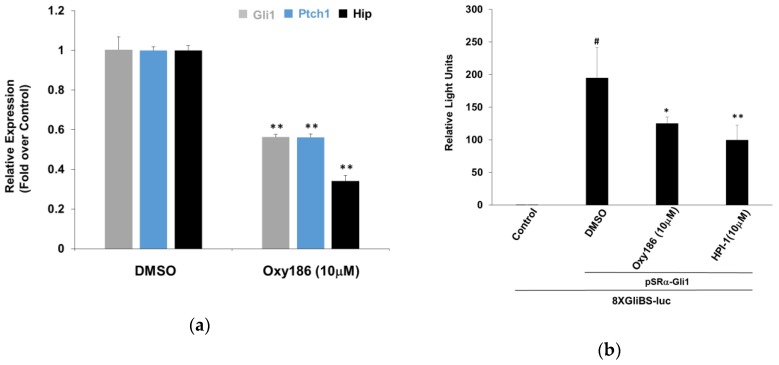

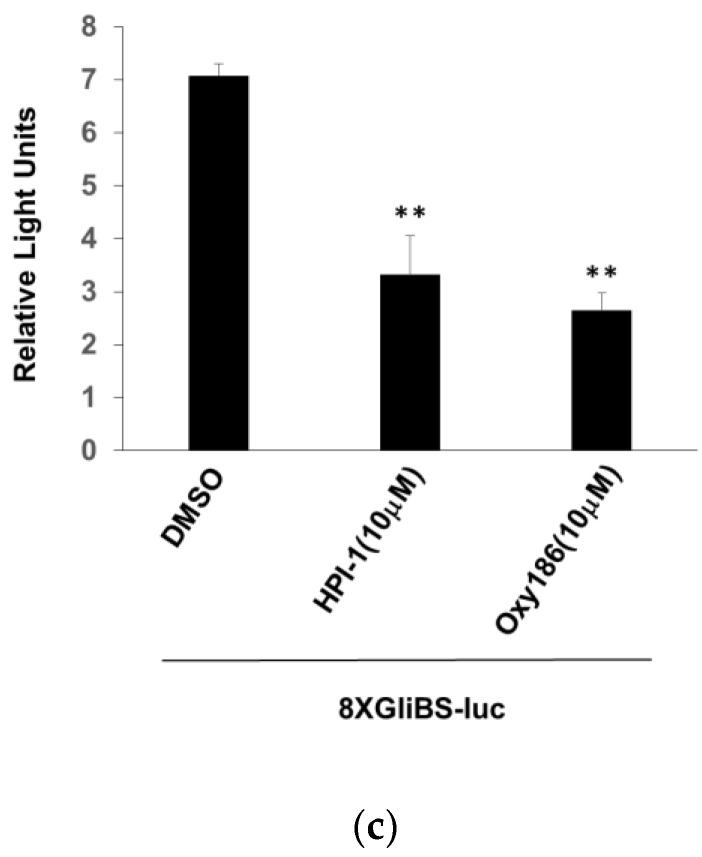
Oxy186 acts downstream of Smo. (**a**) *Sufu*^−/−^ mouse embryonic fibroblast (MEF) cells were treated with DMEM containing 10% FBS in the presence or absence of Oxy186 (10 µM). After 72 h, RNA was extracted and analyzed by Q-RT-PCR for the expression of Hh target genes *Gli1*, *Ptch1*, and *Hip*, normalized to *Oaz1* expression. (**b**) NIH3T3 cells cultured in 24-well plates were transfected with a Gli response-element reporter (pGL3b-8xGliBS-Luciferase) plasmid, a pTK-Renilla-Luciferase plasmid and a vector expressing Gli1, pSRa-Gli1. 24 h after transfection, cells were treated with the test agents as indicated for 72 h. (**c**) *Sufu*^−/−^ cells cultured in 24-well plates were transfected with a Gli response-element reporter (pGL3b-8xGliBS-Luciferase) plasmid and a pTK-Renilla-Luciferase plasmid. 24 h after transfection, cells were treated with the compounds, as indicated, for 72 h. Luciferase activity was measured and normalized to the Renilla luciferase activity. Data from a representative experiment are reported as the mean of triplicate determinations ± SD (* *p* < 0.05 vs. DMSO; ** *p* < 0.01 vs. DMSO; ^#^ *p* < 0.01 vs. control).

Pathogenic Hh pathway activation may occur in cancer at several levels of the Hh signal transduction cascade, including mutations in *Ptch1*, *Smo*, and *Sufu*, as well as amplification of *Gli1* [12,29,43,44,45,46]. Sufu is a negative regulator of Hh signaling, required to process the Gli transcription factors, and its loss results in Smo independent Gli activation [45]. To examine whether oxysterols, such as Oxy186, inhibit Hh signaling epistatic to Sufu, we tested the effect of Oxy186 on *Sufu*^–/–^ mouse embryonic fibroblasts (MEFs). Treatment of *Sufu*^−/−^ MEFs with Oxy186 showed significant inhibition of high baseline expression of Hh pathway target genes *Gli1*, *Ptch1*, and *Hip*, suggesting that Oxy186 exerts its inhibitory effect epistatic to Sufu (Figure 5a). 

### 3.3. Oxy186 Inhibits the Transcriptional Activity of Gli1

Gli1 transcription factors are the effectors of Hh signaling. As direct inhibition of *Gli* expression by siRNA or by the Gli inhibitor Gant61 has been shown to block pancreatic cancer cell growth and survival [40], Gli1 could be considered a ‘druggable’ therapeutic target in the Hh pathway, downstream of Smo [21]. In order to assess the inhibitory effects that oxysterols, such as Oxy186, exert on Hh and Gli signaling, we examined the ability of Oxy186 to inhibit Gli1 transcriptional activity in NIH3T3-E1 cells and found that Oxy186 significantly inhibited the transcriptional activity of a Gli responsive luciferase reporter that was trans-activated by over expressing exogenous Gli1 (Figure 5b). Oxy186 also significantly inhibited the same reporter activity in *Sufu*^−/−^ cells, in which the reporter was trans-activated by endogenous Gli that is constitutively activated due to the loss of Sufu. In *Sufu*^−/−^ cells, Oxy186 exhibited Gli inhibitory effect at 10 µM, comparable to the known Gli inhibitor HPI-1 [31] at the same concentration (Figure 5c).

### 3.4. Oxy186 Inhibits Proliferation of Human Cancer Cells

**Figure 6:**
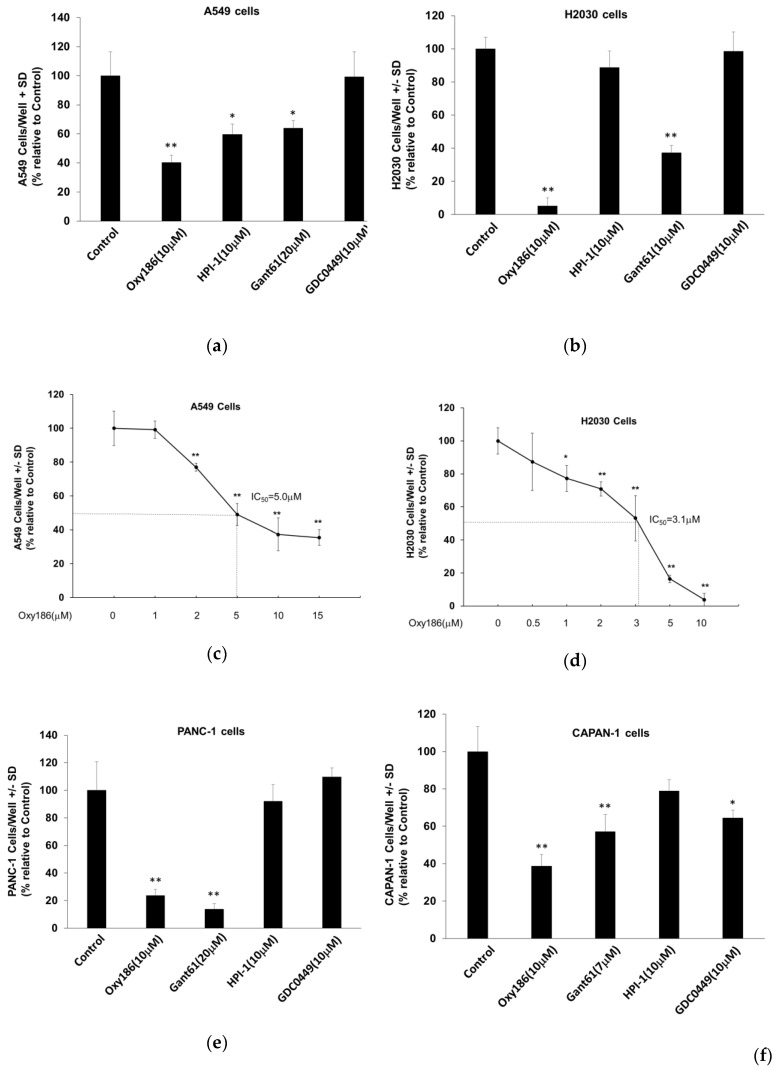
Inhibition of human pancreatic and lung cancer cell proliferation by Oxy186. (**a**,**c**) A549, (**b**) and (**d**) H2030, (**e**) PANC-1, and (**f**) CAPAN-1 cells were treated with the compounds as indicated in RPMI 1640 (**a**–**d**) or DMEM (**e**,**f**) containing 5% FBS for 96 h and then were trypsinized and counted. Data from a representative experiment are reported as the mean of triplicate determinations ± SD (* *p* < 0.05 vs. Control; ** *p* < 0.01 vs. control).

Several reports have linked Hh signaling to upregulated cell proliferation in lung and pancreatic cancer cells [21,40,41,47]. Accordingly, we investigated whether Oxy186 could inhibit cell proliferation in A549 and H2030 NSCLC cells, and in PANC-1 and CAPAN-1 PDAC cells, using cell counting assays. The proliferation of A549 cells was significantly inhibited by Oxy186 and HPI-1 at 10 µM and by Gant61 at 20 µM, but not by vismodegib (GDC0449) at 10 µM (Figure 6a). The IC_50_ of Oxy186 for inhibition of cell proliferation in A549 cells was determined to be 5 µM (Figure 6c). In H2030 cells, Oxy186 and Gant61 displayed a significant anti-proliferative effect (Figure 6b), with an IC_50_ for Oxy186 of 3.1 µM (Figure 6d). In PANC-1 and CAPAN-1 cells significant anti-proliferation effects were observed for Oxy186 and Gant61, but not for HPI-1 (Figure 6e,f). GDC0449 was marginally effective only in CAPAN-1 cells (Figure 6f).

### 3.5. Oxy186 is a Weak LXR Activator

**Figure 7:**
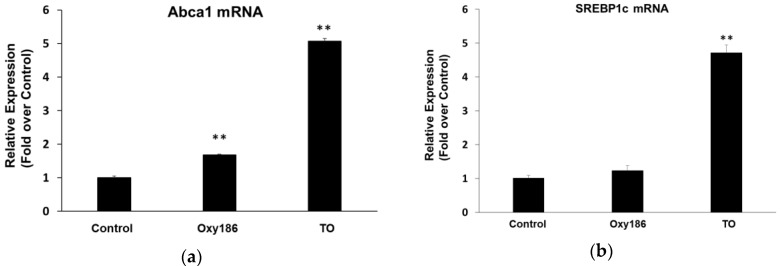
Induction of LXR target gene expression by Oxy186 and LXR agonist TO901317 (TO). (**a**) NIH3T3 cells and (**b**) HepG2 cells were treated with DMEM containing 5% FBS in the presence or absence of TO (2 µM) or Oxy186 (10 µM). After 48 h, RNA was extracted and analyzed by Q-RT-PCR for the expression of LXR target genes *Abca1* in NIH3T3 cells and *SREBP1c* in HepG2 cells and normalized to *Oaz1* or *GAPDH* expression, respectively. Data from a representative experiment are reported as the mean of triplicate determinations ± SD (** *p* < 0.01 vs. control).

Unlike naturally occurring oxysterols previously tested in our laboratory [35], including 20(*S*)-hydroxycholesterol and 22(*R*)-hydroxycholesterol, which activated various levels of both Liver X Receptor (LXR) and Hh pathway activity, Oxy186, tested up to a final concentration of 10 µM, only modestly induced the expression of the LXR target gene, *Abca1*, but not Hh target genes, *Gli1* and *Ptch1*, in NIH3T3-E1 cells (Figure 3a–c and Figure 7a). Treatment of NIH3T3-E1 and HepG2 cells with 2 µM of the non-steroidal LXR agonist, TO901317 (TO), significantly induced the expression of LXR target genes, *Abca1*, and *SREBP1c*, respectively, after 72 h of treatment (Figure 7). In our previously reported studies, activation of LXRs by Oxy16 was found not to be responsible for its inhibition of Hh signaling [37]. Unlike Oxy16, Oxy186 only induced *Abca1* to a modest extent in NIH3T3 cells (Figure 7a), and, unlike non-steroidal LXR agonists such as TO, in the human hepatocyte cell line HepG2, Oxy186 did not induce the expression of *SREBP1c* at 10 µM (Figure 7b). SREBP1c is a master transcriptional regulator of lipogenesis involved in human obesity, type 2 diabetes, and liver steatosis.

### 3.6. Drug-Like Properties of Oxy186

**Figure 8:**
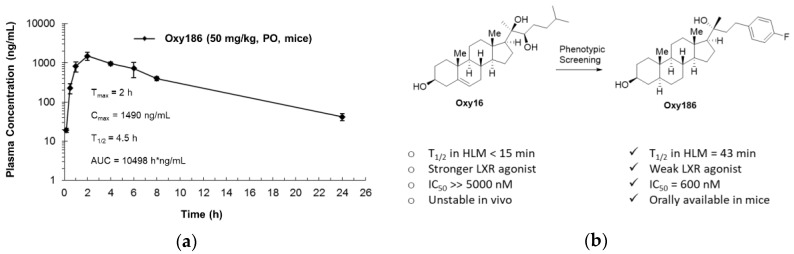
Drug-like properties of Oxy186. (**a**) A single dose of Oxy186 at 50 mg/kg, formulated in 4% ethanol/ 15% DMSO/ 81% corn oil, was administered to balb/c mice by oral gavage. Plasma samples were taken at 10 min, 30 min, 1, 2, 4, 6, 8, and 24 h, followed by LC/MS analysis of the plasma. (**b**) Evolution of Oxy186 from parent compound Oxy16 and their drug-like properties.

In an initial assessment of the drug-like properties of Oxy186, we performed a pharmacokinetic (PK) study in mice. Oxy186, formulated in 4% ethanol, 15% DMSO, and 81% corn oil, was administered to balb/c mice by oral gavage as a single dose of 50 mg/kg. Oxy186 was well tolerated by the mice, showing no overt signs of toxicity. As shown in Figure 8a, Oxy186 displays attractive drug-like absorption and elimination characteristics, with the maximum serum concentration (C_max_) of 1490 ng/mL (3.4 µM, Oxy186 MW = 442.32) observed after 2h (T_max_) and an oral half-life of 4.5 h. The overall exposure, or ‘area under the curve’ (AUC), of 10498 h × ng/mL (23.7 µM × h, suggests that with oral dosing, plasma concentrations can be achieved that will likely fall into a therapeutically meaningful range in mouse disease models.

Additionally, Oxy186 was determined to be more stable than its parent compound Oxy16 when incubated with human and mouse liver microsomes (HLM, MLM), as summarized in Table 1.

## 4. Discussion

In this report we outlined efforts to identify and characterize new sterol-based inhibitors of Hh signaling, exemplified here by Oxy186, that could serve as potential drug candidates. We have studied these new inhibitors using in vitro assays that model cellular signaling that may occur in PDAC stroma, and in pancreatic and NSCLC cells: (1) inhibition of Hh signaling in NIH3T3-E1 fibroblasts treated with conditioned medium (CM) from CAPAN-1 cells containing Hh proteins, a model of paracrine Hh signaling; (2) inhibition of Hh signaling in NIH3T3E1 cells and *Sufu* null mouse embryonic fibroblasts (MEFs), in which Hh signaling is activated by events downstream of Smo; (3) inhibition of endogenous *Gli1* expression in human pancreatic and lung cancer cells, such as PANC-1, A549 and H2030 cells; and (4) inhibition of proliferation in cultures of A549, H2030, PANC-1, and CAPAN-1 cells. 

We previously studied Oxy16 (20-α, 22(*R*)-dihydroxycholesterol), a naturally occurring oxysterol and key metabolite of cholesterol involved in the rate limiting step of steroid biosynthesis [37,49]. When we first identified Oxy16 as an Hh pathway inhibitor, we initially expected that Oxy16 may also be a Smo binder, given the structural similarities (Figure 1a) between Oxy16 and 20(*S*)-hydroxycholesterol (20(*S*)-OHC). 20(*S*)-OHC has been shown to bind Smo and induce allosteric activation of Hh signaling [35,50]. However, further investigation determined the inhibitory mechanism of Oxy16 to be entirely independent of Smo binding [38], and Oxy16 appears to act on a yet unidentified target downstream of Smo and epistatic to Sufu, likely at the level of the transcription factor Gli. In pursuing this new inhibitory mechanism through phenotypic structure activity relationship (SAR) screening and using Oxy16 as a starting point, we identified Oxy186 as a new oxysterol-based chemotype of Hh inhibitors which mechanistically resembles Oxy16 (see also Figure 8b) and acts downstream of Smo to block Hh signaling.

Pioneering studies in Gli1 inhibition have identified Gant61, HPI-1, and JK-184 (Figure 1a) as lead molecules capable of Hh pathway inhibition ‘South of Smo’ [30,31,32]. Gant61, in particular, has been widely embraced as a pharmacologic tool in numerous proof of principle studies, illustrating the benefits of direct Gli1 inhibition in a number of oncologic indications, including solid (pancreatic, ovarian, prostate, breast, lung, and brain cancer) and liquid tumors (multiple myeloma and acute myeloid leukemia) [33]. Beyond these encouraging proof of principle studies, however, the potential for further clinical development of various reported Gants, HPIs, and molecules, like JK-184, remains quite limited for different reasons (limited potency, drug stability, solubility, ADME properties, etc.). New Gli1 inhibitors, with improved drug-like properties and prospects for clinical development, will likely be sought after and embraced by the cancer research and drug development communities. 

Oxy186 caused robust inhibition of ligand-induced and ligand-independent Hh signaling. In NIH3T3 cells activated by Hh ligands, the in vitro potency of Gli inhibition for Oxy186 (Figure 3a–d, IC_50_ = 0.6 µM) clearly exceeded that of Oxy16 (Figure 3a, IC_50_ > 5 µM, unpublished data) and compared favorably with Gant61 and HPI-1 (Figure 3b). In addition, Oxy186 effectively suppressed Hh signaling that resulted from allosteric activation of Smo (38), as shown in Figure 3f. In *Sufu*^−/−^ MEF cells, a model of constitutively activated Gli signaling, Oxy186 significantly inhibits the expression of Hh target genes (Figure 5a) and the endogenous activity of Gli to a similar extent as HPI-1 (Figure 5b,c). Using a luciferase reporter driven by Gli transcriptional activity, as shown in Figure 5b, we found that Oxy186 significantly inhibited the transcriptional activity of Gli1 overexpressed in NIH3T3-E1 cells, suggesting that, like its parent compound Oxy16, Oxy186 acts epistatic to Sufu and possibly at or near the level of Gli, although the direct target of Oxy186 remains unknown at this time. 

When Oxy186 was examined in human cancer cells, we observed significant inhibition of endogenous *Gli1* expression in PANC-1, A549, and H2030 cells (Figure 4a–c), which corresponds well with the anti-proliferative effects of Oxy186 detected in these cells (Figure 6a–e). However, for HPI-1, and to a lesser degree Gant61, inhibition of GLI1 and cell proliferation data do not correlate as smoothly. Unlike Oxy186, HPI-1 displayed significant inhibition of *Gli1* expression but no significant anti-proliferative effects in H2030 and PANC-1 cells (Figure 4c,d, Figure 6b,e), which may suggest that other pharmacological effects exerted by HPI-1 could interfere with its anti-proliferative effects in these cells. Published reports indicate a similar disconnect between Gli1 inhibition and cell proliferation for HPI-1 in T2 breast cancer cells [51]. Conversely, Gant61 displayed significant anti-proliferative effects in PANC-1 cells (Figure 6e), while Gli1 mRNA was only modestly reduced in these cells (Figure 4a), suggesting the possible involvement of other contributing factors, such as nonspecific or toxic effects, for example.

In CAPAN-1 cells, which produce Shh ligand [37] but express very little Gli1 mRNA, treatment with Oxy186, HPI-1, and Gant61 resulted in a significant inhibition of *Shh* expression (Figure 4d), indicating that Shh ligand expression may also be influenced by Hh signaling in some cancer cells. This in turn may help explain the moderate anti-proliferative effects (Figure 6f) observed for Oxy186 in CAPAN-1 cells. In this way, Oxy186 may help attenuate Hh ligand production (in sensitive cancer cells) that stimulates fibroblasts in the surrounding stroma via Hh paracrine signaling and autocrine Hh signaling in tumor cells. It is conceivable, therefore, that oxysterols, such as Oxy186, could produce synergistic inhibitory effects in the tumor microenvironment by modulating both the ligand production in tumor cells (Figure 4d) and the stromal response to ligand in fibroblast cells (Figure 3a–d). 

Tumor stroma can potentially support cancer initiation, progression, and metastasis, but, depending on the context, may also exert some tumor restraining properties [52]. In addition, stromal elements can hold prognostic potential, as documented in in several solid tumors, such as breast, colon, esophageal, lung, and liver cancer [53]. In pancreatic cancer, the stroma-rich nature of the tumor tissue is a very prominent feature, with upwards of 80% of the tumor volume consisting of dense non-cancerous fibrotic scar tissue that encapsulates smaller neoplastic cores [23]. Paracrine Hh signaling emanating from PDAC tumor cells has long been suspected as a contributing factor in the formation and buildup of PDAC associated desmoplasia [7,22]. However, the role of various stromal elements throughout different disease stages has not been fully resolved beyond controversy in PDAC and other stroma rich tumors, such as colon cancer [24,25,42]. Considerable evidence suggests that PDAC-associated stroma may support disease progression by enhancing over-all tumor volume, affecting vascularization and promoting metastasis, both proximal within the pancreas and to distant organs [23,54]. Furthermore, PDAC-associated stroma has been shown to induce resistance to both chemotherapy and radiation by creating a barrier to intra-tumoral drug delivery and shielding cancerous cells from radiation in animal models of pancreatic cancer [55]. By contrast, other publications point to the opposite conclusion by suggesting that stromal elements may act to restrain, rather than support, PDAC progression, based mainly on observations in various transgenic mouse models of the disease [24,56,57,58,59]. In this context, drug-like molecules that effectively block formation and buildup of tumor associated stroma could be valuable research tools, particularly if both ligand production and the stromal response could be targeted. Oxysterols, such as Oxy186, could therefore be useful in the context of a stroma depletion treatment strategy for stroma rich tumors. We plan to examine this possibility in future studies.

As some oxysterols are known endogenous ligands of LXR receptors [60], we routinely monitor oxysterol analogues for LXR activation. However, the Hh inhibitory properties of Oxy16 and Oxy186 appear to be unrelated to LXR activation. Compared to other oxysterols, such as 20(*S*)-OHC and Oxy16, the induction of *Abac1* by Oxy186 appears to be diminished and Oxy186 did not display any detectable induction of *SREBP-1c* in HepG2 hepatocyte cells (Figure 7a,b). The induced expression of *SREBP1c* in liver cells is considered an undesirable off-target effect that contributes to fatty liver formation in vivo and has prevented the therapeutic development of LXR agonists such as TO901317 for clinical applications [61]. These findings add to the apparent safety of oxysterols, such as Oxy186, as potential drug candidates. In terms of PK properties, we determined that Oxy186 is considerably more stable when incubated with human and mouse liver microsomes (HLM and MLM, respectively), in comparison to Oxy16 (Table 1). In addition, Oxy186 displayed attractive drug-like absorption and elimination characteristics when dosed orally in mice (Figure 8a). Taken together, these results suggest that for some Gli inhibition studies previously conducted with Gant61, Oxy186 could emerge as a more suitable drug candidate in the future based on its oral bioavailability and better safety properties. 

In summary, we have identified and characterized Oxy186 as a novel inhibitor of Hh signaling in a cellular context relevant to malignancies with inappropriate pathway activation, which includes a significant subset of human pancreatic and lung cancer. Oxy186 represents a new Hh pathway inhibitor chemotype with promising therapeutic potential as an orally bioavailable drug candidate. Significantly, Oxy186 can inhibit Hh signaling downstream of Smo, in a Smo independent fashion. This is a new and different inhibitory mechanism compared to that of established Smo antagonists, such as vismodegib and sonidegib, whose clinical efficacy is often limited by Gli activation that occurs downstream of Smo. In future studies, we plan to examine the efficacy of Oxy186 and related analogues in human patient-derived xenograft models and will correlate morphological findings and histological observations with the suppression of Hh signaling in such tumor specimens.

## Figures and Tables

**Table 1:** Metabolic stability in human and mouse liver microsomes (HLM and MLM, respectively).

Compound ^1^	HLM, % Remaining after 60 min	HLM, Half-Life	MLM, % Remaining after 60 min	MLM, Half-Life
Oxy16	6%	15 min	5%	14min
Oxy186	40%	45 min	50%	60min

^1^ Metabolic stability testing was performed at Eurofins, Inc., according to methods described by Obach et al. [48]. The drug-like properties of Oxy186 are summarized in Figure 7a,b.
